# Monte Carlo modelling and validation of the elekta synergy medical linear accelerator equipped with radiosurgical cones

**DOI:** 10.1016/j.heliyon.2023.e15328

**Published:** 2023-04-14

**Authors:** P.S. Renil Mon, V.N. Meena-Devi, Saju Bhasi

**Affiliations:** a Department of Physics, Noorul Islam Centre for Higher Education, Kumarakoil, Kanyakumari District, Tamilnadu, India; b Department of Radiation Physics, Regional Cancer Centre, Thiruvananthapuram, Kerala, India

**Keywords:** Monte Carlo method, Gamma analysis, Medical linear accelerators, Radio surgical cones, Dose profile

## Abstract

Monte Carlo simulations of medical linear accelerator heads help in visualizing the energy spectrum and angular spread of photons and electrons, energy deposition, and scattering from each of the head components. Hence, the purpose of this study was to validate the Monte Carlo model of the Elekta synergy medical linear accelerator equipped with stereotactic radio surgical connical collimators. For this, the Elekta synergy medical linear accelerator was modelled using the EGSnrc Monte Carlo code. The model results were validated using the measured data. The primary electron beam parameters, beam size, and energy were tuned to match the measured data; a dose profile with a field size of 40 × 40 cm^2^ and percentage depth dose with a field size of 10 × 10 cm^2^ were matched during tuning. The validation of the modelled data with the measurement results was performed using gamma analysis, point dose, and field size comparisons. For small radiation fields, relative output factors were also compared. The gamma analysis revealed good agreement between the Monte Carlo modeling results and the measured data. A gamma pass rate of more than 95% was obtained for field sizes of 40 × 40 cm^2^ to 2 × 2 cm^2^ with gamma criteria of 1% and 1 mm for the dose difference (DD) and distance to agreement (DTA), respectively; this gamma pass rate was more than 98% for the corresponding values of 2% and 2 mm for the DD and DTA, respectively. A gamma pass rate of more than 99% was obtained for a percentage depth dose with 1 mm and 1% criteria. The field size was also in good agreement with the measurement results, and the maximum deviation observed was 1.1%. The stereotactic cone field also passed this analysis with a gamma pass rate of more than 98% for dose profiles and 99% for the percentage depth dose. The small field output factor exhibited a deviation of 4.3%, 3.4%, and 1.9% for field sizes of 5 mm, 7.5 mm, and 10 mm, respectively. Thus, the Monte Carlo model of the Elekta Linear accelerator was successfully validated. The validation of radio surgical cones passed the analysis in terms of the dose profiles and percentage depth dose. The small field relative output factors exhibited deviations of up to 4.3%, and to resolve this, detector-specific and field-specific correction factors must be derived.

## 1 Introduction

Megavoltage-photon-beam linear accelerators are widely used in radiation therapy treatments. Linear accelerators produce mega-electron-volt energy photons and electron beams, which are used in cancer treatments. An accurate calculation of the radiation dose deposited in a patient's body is important in radiotherapy. Treatment success and normal tissue complications are directly related to the accuracy of dose deposition. The dose calculation accuracy is an integral part of therapeutic gain [[1], [2], [3]]. A dose difference of 7% has been reported to be clinically detectable [4]. Additionally, a 5% change in the dose has been reported to affect the Tumour control probability (TCP) by up to 20% and the Normal tissue complication probability (NTCP) by up to 30% [[5], [6], [7]]. Treatment planning systems (TPSs) determine the dose distribution in the patient geometry based on commercial dose calculation algorithms. Some of these algorithms are based on the Monte Carlo (MC) calculation method, and others use the MC-calculated spectrum and dose kernels [8].

The MC simulation is used in various fields of science and technology. It is a statistical technique adopted to solve multi-dimensional integral equations based on random numbers. The MC simulation is important when no analytical solution can be obtained, or the accuracy of the analytical solution is beyond tolerance [9]. The quick calculation speeds of modern computers have made MC simulations possible in clinical settings. In radiation transport simulations, possible interaction processes are simulated according to the probability distribution of each interaction process. As the physics of each interaction process is well known, MC simulations are fairly straightforward [10]. In radiation transport simulation, the trajectory of each particle is simulated, and such particle trajectories are referred to as histories. The fate of the particle is determined by the set cut-off energy or geometric boundary of the simulation. The quantities of interest are obtained as averages over the number of histories. The statistical accuracy of the simulation result depends on the number of histories simulated. The number of histories required to achieve a specified statistical accuracy depends on the property of interacting particles and the nature of the interaction process.

The MC simulation of a medical linear accelerator head helps in visualizing the energy spectrum and angular spread of photons and electrons, energy deposition, and scattering from each of the head components. Particle transport through complicated beam-shaping devices, like Multi-leaf collimators (MLC) and stereotactic cone shape collimators, is extremely important in clinical dosimetry, such as the verification of the Dosimetry leaf gap (DLG), tongue and groove effect, calculation of small field correction factors, and electron contamination in photon fields [[11], [12], [13]]. Particle transport is also used to calculate the ionization chamber-dependent correction factors for reference dosimetry. The calculation of deposited doses in heterogeneous tissues like lung and dense bones is still challenging for most commercial planning systems owing to complex approximations [14]. With a validated accelerator head model, the dose distribution in computed tomographic images can be obtained and compared with the TPS determined dose distribution; this can be considered as a secondary check in clinics [15]. Therefore, the implementation of the validated MC model of a clinical medical linear accelerator is crucial.

In this study, we modelled the Elekta synergy linear accelerator equipped with radio surgical conincal collimators using the BEAMnrc version 2.0 MC code, and the model was then validated for beams with energies of 6 MV based on measurements conducted in water. Separate validation was performed for the dosimetric characteristic of Stereotactic radio surgical (SRS) collimators. Finally, the MC dose calculation was accomplished by using the DOSEXYZnrc code.

## 2 Materials and methods

### 2.1 BEAMnrc simulation

BEAMnrc is a MC code for linear accelerator head simulation belonging to the (Electron Gamma Shower (EGSnrc)) system of computer codes. EGSnrc is a general-purpose MC code, which can simulate the coupled transport of electrons, positrons, and photons through an arbitrary geometry in an energy range of a few keV to several hundred GeV. The Photon Electron Gamma Shower (PEGS4) code generates data from a cross section table of elements for the EGSnrc simulation [16]. To reduce the calculation time, EGSnrc uses a Condensed history (CH) technique for electron transport. Generally, high energy electrons undergo numerous collisions during transport in the medium, which slows down the simulation process. Therefore, in CH techniques, numerous subsequent interactions of electrons are considered as a single step. The effect of individual interactions is considered by sampling the direction, energy, and position at the end of the step based on the multiple scattering distribution [16,17].

The BEAMnrc simulation of the Elekta synergy linear accelerator is conducted based on the recommendations of AAPM TG 105 guidelines [10]. The BEAnrc simulation is executed in two parts. The first part is initiated from the X-ray target; thereafter, the simulation proceeds through the primary collimator flattening filter, monitor chamber, and mirror, and ends with a phase space file scored at a distance of 27.21 cm from the target. The second part of the simulation is initiated from the MLC and proceeds through the X and Y jaws and a thin mylar sheet, which defines the crosswires. This part of the simulation uses the phase space file generated by the first part as the input source and scores the second phase space file at a distance of 100 cm from the target. BEAMnrc component modules are used to define the geometry of each of the beam line components. The electron cut-off energy (ECUT) energy used for the simulation is 0.7 MeV, and the (photon cutoff energy) PCUT energy is 0.01 MeV. Note that the same cut-off energies are used for the PEGS4 data file creation. The variance reduction techniques used in the BEAMnrc simulation include range rejection and uniform bremsstrahlung splitting [18]. All other parameters of the BEAMnrc are set to their default values. The number of histories used in each simulation is 10^8^, and all simulations are performed using a 12 core Dell T5600 workstation.

The primary electron beam source is defined with the BEAMnrc source number 19, which is an elliptical source with a gaussian distribution along the X,Y, parallel, and angular spread [18]. The source parameters, full width at half maximum (FWHM), and energy are tuned to match with the measured profile and percentage depth dose (PDD). As the off-axis ratios are more sensitive to the FWHM [19], the tuning of the FWHM initiates with the comparison of a 40 × 40 cm^2^ field off-axis ratio with the measured values. During the tuning, the FWHM value changes from 0.7 mm to 1.3 mm. Moreover, the central axis depth dose is more sensitive to the primary beam electron energy [19]. Therefore, the primary photon beam energy is tuned by comparing the PDD corresponding to a 10 × 10 cm^2^ field size with the measured value. During the tuning, the electron beam energy changes from 5.8 MeV to 6.4 MeV. The off-axis ratios also depend on the primary electron energy. Therefore, during each energy variation, off-axis profiles with sizes of 40 × 40 cm^2^ are also compared with measured values.

After the tuning of the primary electron beam FWMM and energy, the second part of the simulation is repeated with field sizes of 40 × 40 cm^2^, 30 × 30 cm^2^, 25 × 25 cm^2^, 20 × 20 cm^2^, 15 × 15 cm^2^ 10 × 10 cm^2^, 6 × 6 cm^2^, 2 × 2 cm^2^. A phase space file for each field size is saved separately for use as the input source for the DOSXYZnrc simulation.

### 2.2 SRS cone modelling

After obtaining the phase space file for the above field sizes, the second part of the model is modified by fixing the secondary collimator opening to 5 × 5 cm^2^ at a distance of 100 cm from the primary source, and an SRS cone is added to the lower end of the accelerator head. This part of the simulation is repeated for SRS cones with nominal diameters of 15 mm, 12.5 mm, 10 mm, 7.5 mm, and 5 mm. The phase file is scored at a distance of 100 cm from the target for DOSXYZnrc calculations.

### 2.3 DOSXYZnrc simulation

DOSXYZnrc is an MC code belonging to EGSnrc, which determines the three-dimensional dose distribution in a voxelized geometry [20]. In this study, we used this code for PDD and off-axis profile calculation. The MC parameters and cut-off limits were set to the same values as those in the BEAMnrc simulation. The PEGS4 data file obtained for the BEAMnrc simulation was used for DOSXYZnarc. Range rejection of a secondary electron, with 0.7 MeV as the cut-off limit, was selected as the variance reduction technique. The phase space file scored in the second part of the simulation with varying field sizes was used as the input source for the DOSXYZnrc simulation. The number of histories used was 10^9^; this was done to maintain the MC uncertainty below 0.5%. The dose was scored using 1 × 1 × 1 mm^3^ voxels in a 50 × 50 × 50 cm^3^ water geometry for each field size for PDD and off-axis profile calculation.

### 2.4 Measurements

All the measurements were performed on the Elekta synergy accelerator using a 63.5 × 54.5 × 53 cm^3^ water phantom (PTW RFA MP3 3D). In-plane, cross-plane, off-axis profiles, and the PDD were scanned using the PTW Mefisto MC^2^ V3.2 software. All the scans were performed with a resolution of 1 mm to match the MC calculation. A PTW Semiflex ionization chamber with a cavity volume of 0.25 cc was used for all the PDDs and off-axis profiles. The off-axis profiles were analyzed at three different depths: 1.5 cm, 5 cm, and 10 cm, and the results were compared with the MC-calculated profiles. Comparisons between the measured and MC-calculated profiles and PDDs were performed using one dimensional gamma evaluation.

#### 2.4.1 Small field measurement

Small field measurements were done based on the recommendations of IAEA TRS report 483 [21]. Generally, small field measurements are highly uncertain owing to the lack of a charged particle equilibrium, the volume averaging effect, a density perturbation, and positioning error [[22], [23], [24], [25]]. Therefore, the use of detectors with smaller active volumes is recommended [26]. In this study, we used an IBA stereotactic field detector SFD; its active volume was 0.6 mm in diameter and 0.06 mm in thickness. The SFD is a p-type silicon unshielded diode detector specifically used in small field measurements. The PDD, dose profile, and Relative Output factor (ROF) were measured using the SFD. All measumement were done in PTW RFA MP3 3D. Note that the ROF is the ratio of the dose measured at a reference depth with a given field size to the dose measured with reference field size at the same depth. Here, the SRS cone fields had dimensions of 5 mm–15 mm, the reference field had a dimension of 10 × 10 cm^2^, and the reference depth was 1.5 cm, which denotes the depth corresponding to the dose maximum d_max_ of the 6 MV beam. Detector centring was done separately for each collimator by taking inplane and crossplane profiles at the reference depth. After the centring detector charge collection was taken using PTW MP3 Tandum electrometer. For each collimator, charge collection was measured five times and average reading was taken for ROF calculation as the ratio of average charge collected for the conical collimator to the average charge collected for the 10 × 10 cm^2^ field.

### 2.5 Validation

#### 2.5.1 Gamma analysis

**Fig. 1:**
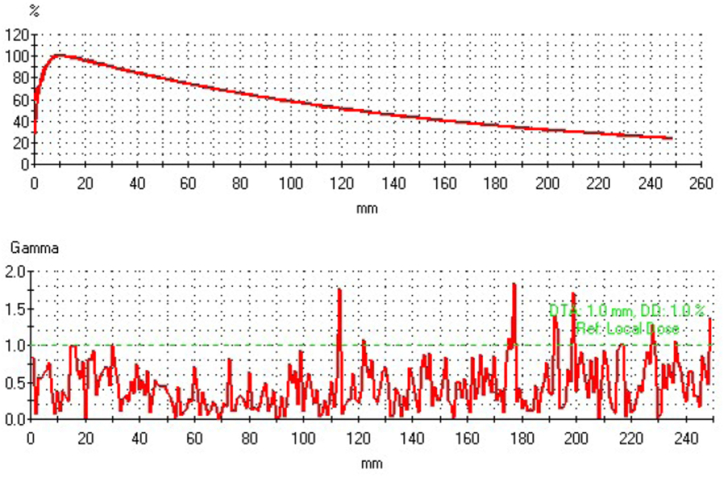

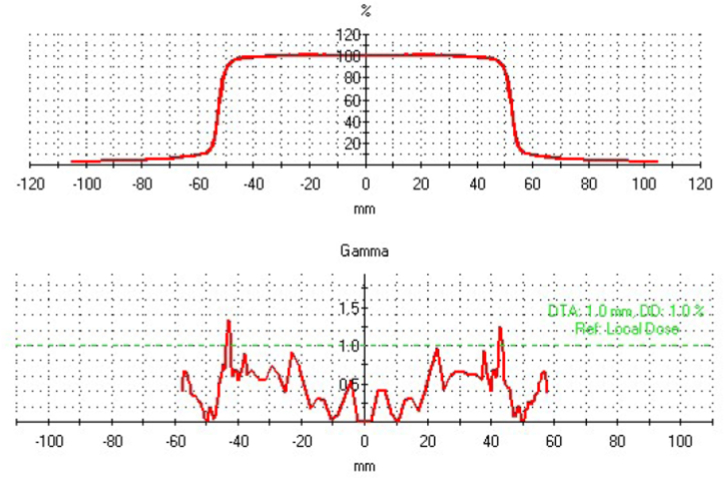
(a) Gamma analysis of the PDD field size 10 × 10 cm^2^: The upper curves shows overlay of measured and Monte Carlo calculated PDDs and gamma values below. (b).Gamma analysis of a dose profile: field size 10 × 10 cm^2^, The upper curves shows overlay of measured and Monte Carlo calculated beam profiles and gamma values below.

A comparison between the MC-calculated and measured data was performed using gamma analysis [27]. Note that the gamma analysis provides a quantitative comparison of a two-dose distribution based on the dose difference (DD) and distance to agreement DTA. The DTA denotes the distance between a data point in the given distribution and the nearest point in the reference dose distribution of the same dose. The gamma analysis compares each data point in the MC-calculated dataset with the points in the measured data set. One MC data point is considered to pass the analysis if its corresponding data point exists in the measured data set with a given DD within a given DTA. The gamma value can be mathematically represented as.

$$\Gamma =\sqrt{{\left(\frac{{\Delta d}_{i}}{\Delta D}\right)}^{2}+{\left(\frac{{\Delta s}_{i}}{\Delta S}\right)}^{2}}$$

where ${\Delta d}_{i}$ denotes the DD between the measured and MC-calculated values at the i_th_ data point, and ${\Delta s}_{i}$ represents the DTA between the two datasets at the i_th_ data point. ΔD and ΔS denote the acceptable limits set for the DD and DTA. If the *Γ* value is less than or equal to one, the corresponding data point is considered to pass the analysis; otherwise, it is considered to fail. In this study, we considered a result to pass the gamma analysis if 95% of the data points in the dataset were found to pass the analysis for two sets of DD and DTA values, which were set to 2% and 2 mm and 1% and 1 mm, respectively. The gamma analysis was conducted by using the PTW Mefisto MC^2^ data analysis software. Fig. 1a and b presents an example of the gamma analysis for a PDD and dose profile.

#### 2.5.2 Point dose and field size comparison

The MC-calculated PDDs at depths of 5 cm, 10 cm, 20 cm, and 30 cm were compared with the measurement results, and the percentage differences were reported. In the dose profile, the field size was calculated as the lateral distance between points at a 50% decrease in the dose. PTW Mefisto MC^2^ data analysis software was used for the field size calculation.

## 3 Results and discussion

### 3.1 Primary electron beam parameters

**Fig. 2:**
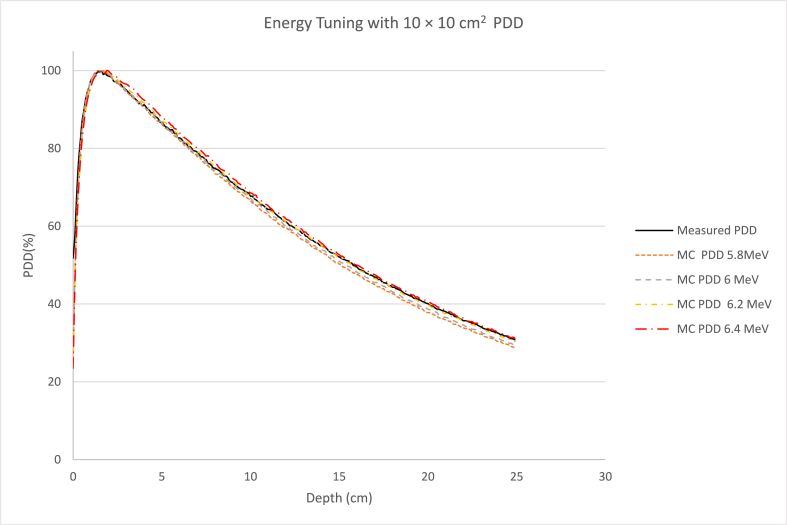
PDD for varying primary beam energy (5.8 MeV–6.4 MeV), field size 10 × 10 cm^2^.

As the FWHM of a beam has negligible effect on the DD, the primary electron beam parameter tuning was initiated with an energy ranging between 5.8 MeV and 6.4 MeV [28]. It is known that as the primary electron energy increases, the mean energy of bremsstrahlung photons also increases, which, in turn, increases the depth dose beyond the build-up region. A perfect match between the measured and MC-calculated PDDs is obtained for a primary electron beam energy of 6.2 MeV. Fig. 2 illustrates the PDD curves corresponding to energy tuning.

**Fig. 3:**
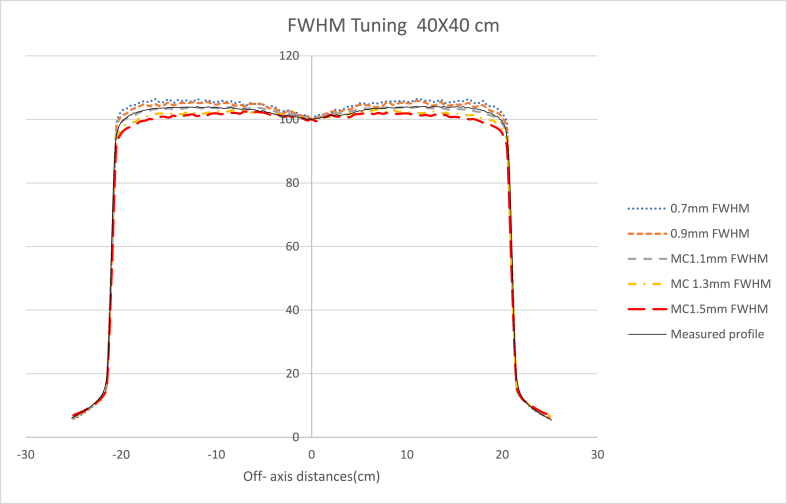
Dose profile of 40 × 40 cm^2^ with varying FWHM (0.7–1.5 mm) and dose profile measured at a depth of 5 cm.

Notably, a change in the primary beam FWHM affects the flatness of the beam. As the FWHM of the primary beam increases, the dose profile appears increasingly flattened. During FWHM adjustment, a perfect match with the measured data is obtained at 1.1 mm. Further increments in the FWHM make the beam profile appear even flatter; finally, the value that is most comparable to the measured value is considered as the final tuned value of the primary electron beam energy. Fig. 3 compares the MC-calculated dose profiles recorded in water for different primary electron FWHMs with the measured dose profile. Note that previous studies conducted by Bagheri and Rogers on the Elekta linear accelerator have reported values of 6.3 MeV and 1.1 mm for the electron beam energy and FWHM, respectively [19]. In another study conducted by Cranmer-Sargison et al. on the Varian iX linear accelerator, the corresponding values were found to be 6.2 MeV and 1.1 mm [29].

### 3.2 Comparison of dose profiles and PDD

**Fig. 4:**
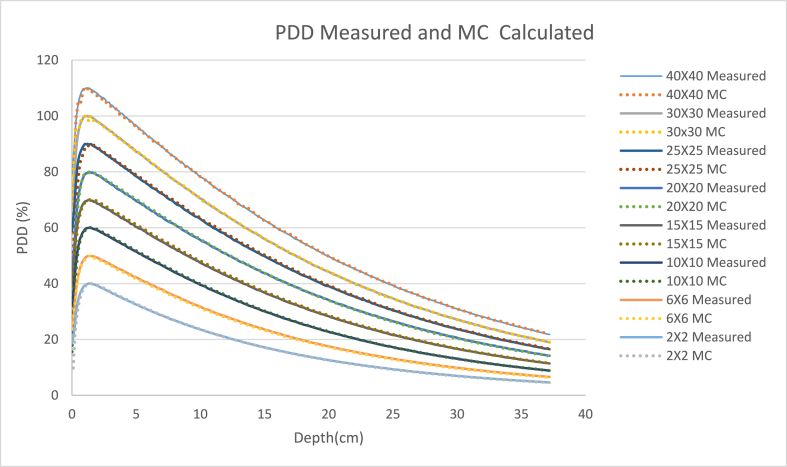
MC-calculated PDD and measured PDD for field sizes of 2 × 2 cm^2^ to 40 × 40 cm^2^.

Further, MC-calculated PDDs and dose profiles for field sizes of 2 × 2, 6 × 6, 10 × 10, 15 × 15, 20 × 20, 25 × 25, 30 × 30, and 40 × 40 were compared with the measurement results. The comparison results between the MC-calculated and measured PDD curves passed the following two gamma criteria: DTA of 2 mm and DD of 2% and DTA of 1 mm and DD of 1% with passing rates of more than 100% and more than 99%, respectively. Failure points were noted only in the build-up region. In the build-up region, a mismatch was found between the MC calculation and measurement results; this mismatch can be primarily attributed to the volume averaging of the dose in the active volume of the detector and lack of electronic equilibrium [[30], [31], [32]]. Fig. 4 illustrates the MC-calculated and measured PDDs plotted for different field sizes.

**Fig. 5:**
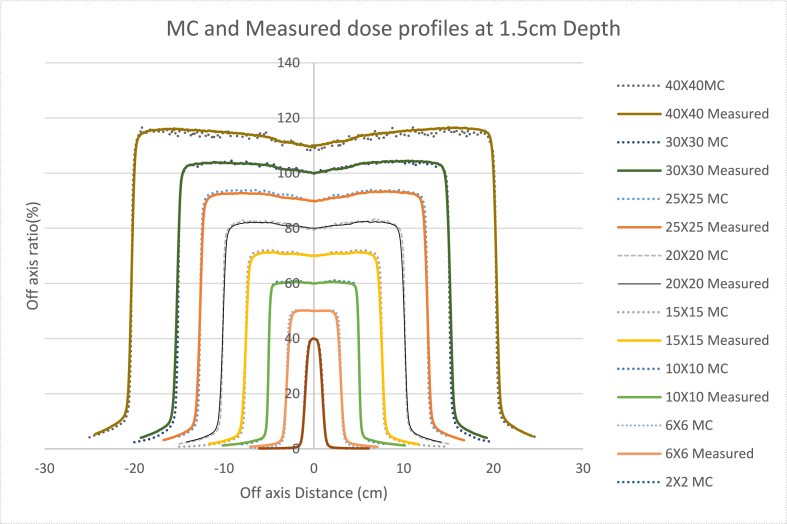

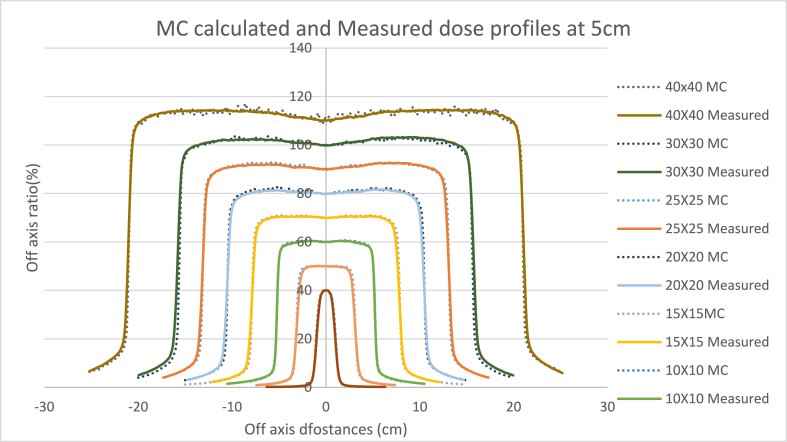

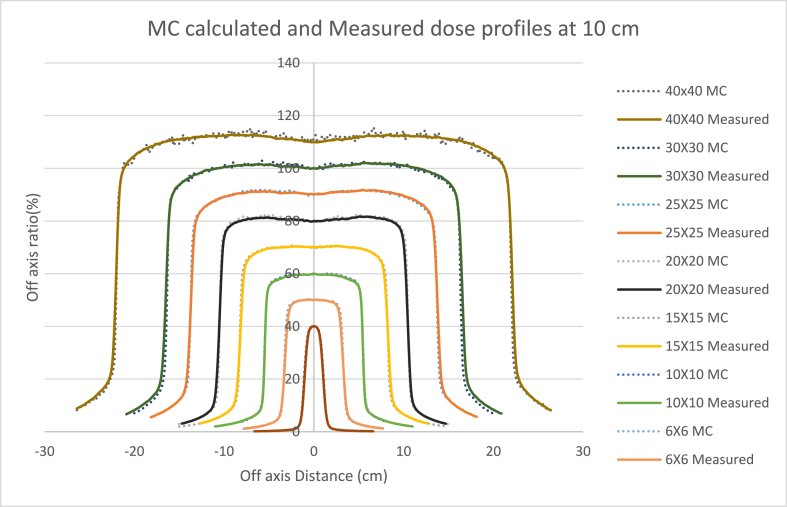
(A)MC-calculated and measured dose profiles at a depth of 1.5 cm for field sizes of 2 × 2 cm^2^ to 40 × 40 cm^2^. (b)MC-calculated and measured dose profiles at a depth of 5 cm for field sizes of 2 × 2 cm^2^ to 40 × 40 cm^2^. (c)MC-calculated and measured dose profiles at a depth of 10 cm for field sizes ranging from 2 × 2 cm^2^ to 40 × 40 cm^2^.

The dose profiles were compared at three different depths: 1.5 cm, 5 cm, and 10 cm; the results passed the following gamma criteria: DTA of 2 mm and DD of 2% and DTA of 1 mm and DD of 1% with passing rates of more than 95% and 97%, respectively. Failure points were primarily located in the penumbra of the dose profile; this could be attributed to the volume averaging effect and position tolerance of the collimating jaws [[33], [34], [35], [36], [37]]. The foregoing effect was significant for smaller field sizes. The comparison results of the field size at the profile depth were also in good agreement with measurement results, with a maximum deviation of only 1.08%. Table 1 lists the results of field size comparison and gamma analysis. Fig. 5 illustrates a comparison between the dose profiles measured at the three depths.

**Table 1:** Comparison of MC-calculated and measured profile and field sizes, and the gamma analysis results.

Depth (cm)		Field size at scan depth (cm)				
	Field Size (cm^2^)	MC	Measured	% Difference	Γ(1 mm, 1%) pass rate	Γ(2 mm, 2%) pass rate
1.5	2 × 2	2.02	2.03	−0.49	95.13	98.02
	6 × 6	6.09	6.1	−0.16	97.1	98.5
	10 × 10	10.14	10.11	0.30	96.5	97.67
	15 × 15	15.23	15.22	0.07	96.05	98.6
	20 × 20	20.4	20.35	0.25	97.3	98.7
	25 × 25	25.38	25.43	−0.20	96.2	97.9
	30 × 30	30.36	30.53	−0.56	99.05	100
	40 × 40	40.75	40.78	−0.07	96.29	100
5	2 × 2	2.1	2.09	0.48	96.13	97
	6 × 6	6.35	6.32	0.47	98.2	100
	10 × 10	10.51	10.46	0.48	97.67	100
	15 × 15	15.77	15.75	0.13	97.36	98.68
	20 × 20	21.03	21.08	−0.24	98.3	99.91
	25 × 25	26.26	26.32	−0.23	97.87	99.45
	30 × 30	31.27	31.61	−1.08	98.1	100
	40 × 40	42.02	42.19	−0.40	98.47	99.2
10	2 × 2	2.2	2.21	−0.45	98.03	100
	6 × 6	6.63	6.62	0.15	97.8	99.4
	10 × 10	11.01	10.97	0.36	96.5	100
	15 × 15	16.53	16.51	0.12	96.05	99.3
	20 × 20	22.03	22.06	−0.14	99.3	99.8
	25 × 25	27.5	27.57	−0.25	98.4	100
	30 × 30	32.81	33.1	−0.88	99.5	100
	40 × 40	44.06	44.22	−0.36	99.6	100

### 3.3 SRS cone PDD and profile comparison

**Fig. 6:**
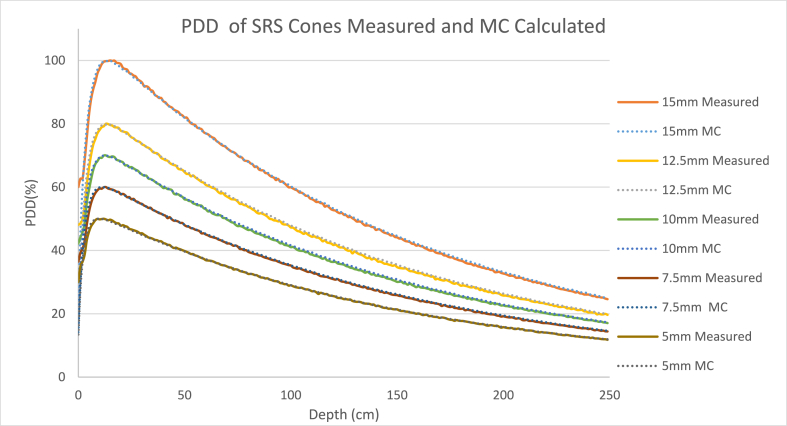
MC-calculated and measured PDDs of SRS cones.

The MC-calculated PDD values were found to be in good agreement with the measured data; the gamma pass rate was more than 95% for DD and DTA values of 1% and 1 mm, respectively, and more than 97% for DD and DTA values of 2% and 2 mm, respectively. Here, failure points were noted in the build-up region. The point doses at 5 cm, 10 cm, and 20 cm were in good agreement with the measurement results. The maximum variation observed was 1.5%. In Fig. 6, the PDDs of all cones, MC-calculated PDDs, and measured PDDs have been plotted.

**Fig. 7:**
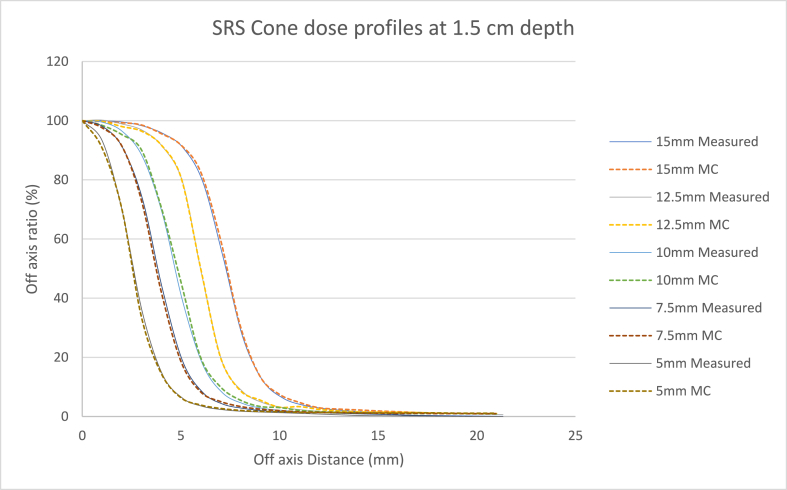

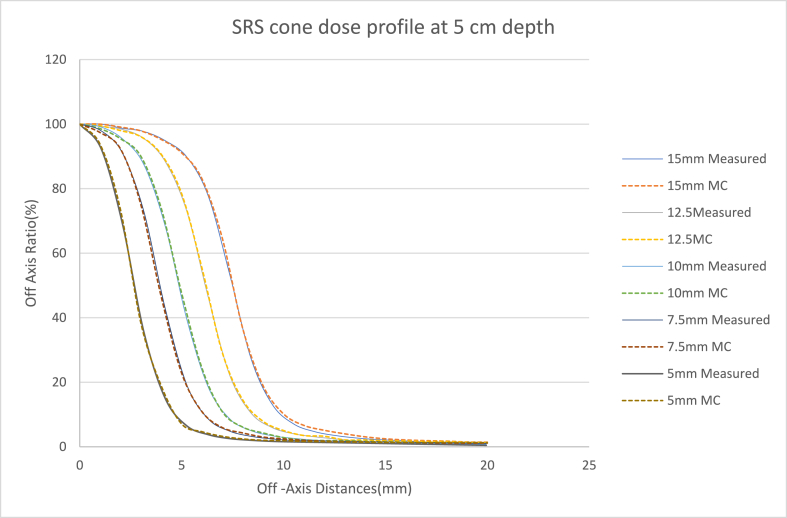

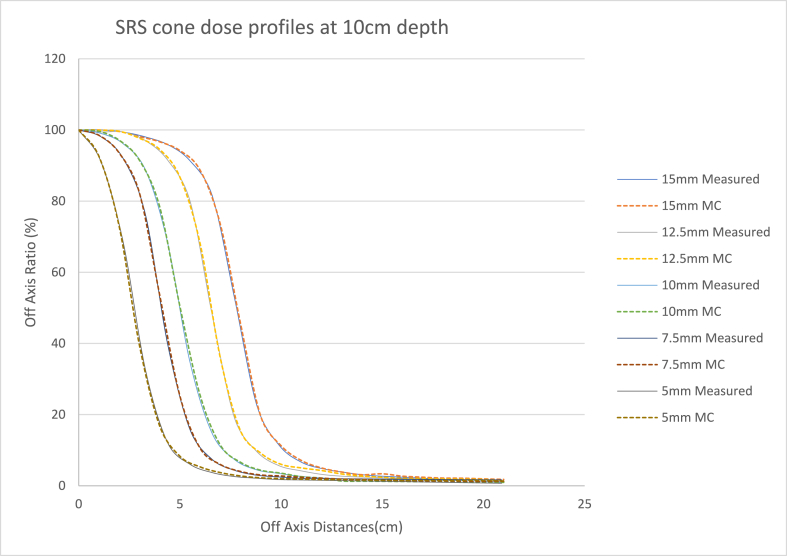
(A). MC-calculated and measured dose profiles at a depth of 1.5 cm. (b). MC-calculated and measured dose profiles at a depth of 5 cm. (c)MC-calculated and measured dose profiles at a depth of 10 cm.

Further, the dose profiles obtained from MC calculations and measurements at depths of 1.5 cm, 5 cm, and 10 cm were compared in terms of the field size using gamma analysis. The MC calculation results appeared to be in good agreement with the measurement results. The passing rate was more the 98% for DD and DTA values of 1% and 1 mm, respectively. The field size differences were all below 1%. The measurement uncertainties associated with the small field were not reflected in the profile and the PDD. As we used the SFD with a small active volume of 0.036 mm^3^, no observable difference was noted in the penumbra. Table 2 lists the results of field size comparison and the gamma analysis. Fig. 7a–c illustrates the comparison between the MC-calculated and measured dose profiles at depths of 1.5 cm, 10 cm, and 20 cm.

**Table 2:** Gamma analysis results for the MC-calculated and measured dose profiles, and comparison of field sizes for SRS cones.

Depth (cm)	Nominal Field Diameter (mm)	Field size at scan depth			Γ(1%, 1 mm) Pass rate %
		MC	Measured	% Difference	
1.5	5	5.22	5.21	0.19	98.3
	7.5	7.67	7.65	0.26	98.6
	10	9.61	9.65	−0.41	99.3
	12.5	11.99	12.01	−0.17	99.8
	15	14.56	14.69	−0.88	100
5	5	5.39	5.34	0.94	99.2
	7.5	7.85	7.89	−0.51	99.4
	10	9.74	9.82	−0.81	100
	12.5	12.32	12.41	−0.73	99.3
	15	14.99	15.04	−0.33	100
10	5	5.53	5.57	−0.72	98.8
	7.5	8.28	8.26	0.24	99.1
	10	10.56	10.54	0.19	100
	12.5	13.06	13.12	−0.46	99.2
	15	15.6	15.68	−0.51	99.7

### 3.4 Comparison of ROF

**Fig. 8:**
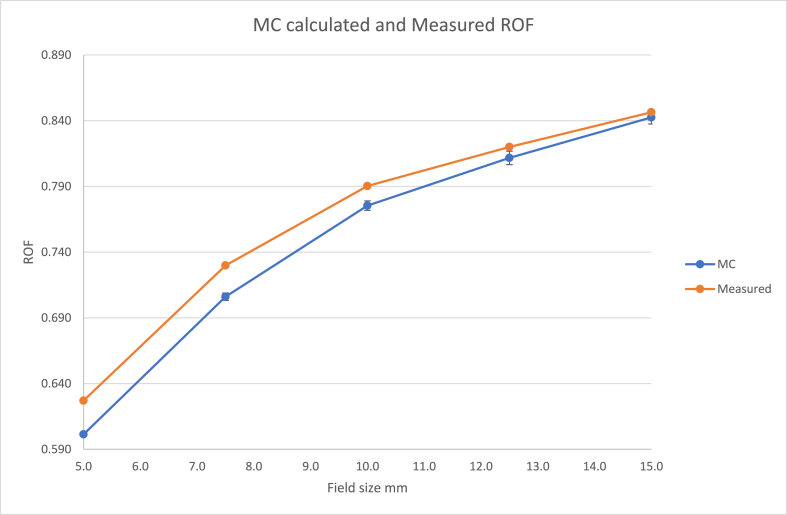
MC-calculated and measured ROF.

A comparison between the MC-calculated and measured ROFs revealed a significant difference at smaller field sizes of 5 mm, 7.5 mm, and 10 mm; the corresponding differences were 4.3%, 3.4%, and 1.9%, respectively. This can be attributed to the density perturbation effect in the silicon chip active volume. As no charged particle equilibrium exists in small fields, the density perturbation effect is significant. In the ROF measurement, an adequate charged particle equilibrium exists in the reference field with dimensions of 10 × 10 cm^2^; hence, the density perturbation here is not significant. As the ROF denotes the ratio computed based on the reference field size, density perturbation leads to an overestimation of the measured ROF, causing a variation in the ROF of up to 4.3% for the smallest field size of 5 mm. This over estimation is not significant in the PDD measurement because the PDD represents the ratio of the dose measured at d_max_ in the same field size. Alfonso et al. [38] proposed a formalism to calculate correction factors for small ROFs based on the MC calculation. Following this, several authors calculated correction factors for different sets of detectors [29,[39], [40], [41], [42], [43]]. These correction factors were detector, collimator, and machine specific. Table 3 and Fig. 8 present the ROFs for all SRS cones. TRS report 483 provided small field correction factors for IBA SFD detector corresponding to the equivalent square field which can be applied for flattened and unflatten beams. The concept of equivalent square is uncertain in very small fields ≤10 mm where the source occlusion is a dominating factor [44]. Bassinet, C. et al. in their work, calculated the MC based correction factors for SFD for Varian Novalis accelerator. For 4 mm Brain lab circular collimator in Novalis accelerator, correction factor was 0.975 [24]. Cheng et al. in their paper reported the difference between measured and MC calculated ROF of SFD for flattening filter free 6 MV beams of Varian Truebeam machine as 3.24%, 2.22%,2.41% for 5 mm,7.5 mm, 10 mm conical collimators respectively [45].

**Table 3:** MC-calculated and measured ROF.

Nominal Field diameter (mm)	MC ROF	Measured ROF	% Difference
5.0	0.601 ± 0.001	0.627	−4.3
7.5	0.706 ± 0.003	0.730	−3.4
10.0	0.775 ± 0.004	0.790	−1.9
12.5	0.812 ± 0.005	0.820	−1.0
15.0	0.842 ± 0.005	0.846	−0.5

## 4 Conclusion

In this study, the MC model of the Elekta linear accelerator equipped with SRS cones was developed and validated with measurements. The MC-calculated dose profiles and PDDs were compared using gamma analysis and point dose comparisons, and the modeling results were found to agree with the measurement results. Small field ROFs demonstrated significant variations of 4.3, 3.4%, and 1.9% for field sizes of 5 mm, 7.5 mm, and 10 mm, respectively. Hence, determining the correction factors for SFD in this set of collimators is essential.

## Funding sources

There is no financial support or funding for this study.

## Author contribution statement

Renil Mon P S: Conceived and designed the experiments; Performed the experiments; Wrote the paper. Meena Devi V N: Analyzed and interpreted the data. Saju Bhasi: Contributed reagents, materials, analysis tools or data.

## Data availability statement

Data included in article/supp. material/referenced in article. Declaration of interest's statement: The authors declare no competing interests.
